# Diagnostic validity of fine-needle capillary cytology in palpable tumours at the Oncology Institute of Peru

**DOI:** 10.3332/ecancer.2018.805

**Published:** 2018-02-01

**Authors:** Milagros Abad-Licham, Jose Galvez-Olortegui, Juan Astigueta, Juan Díaz-Plasencia

**Affiliations:** 1 Graduate School, Antenor Orrego Private University, Trujillo 13007, Peru; 2 Pathology Department, Regional Institute of Neoplastic Diseases, Trujillo 13601, Peru; 3 Scientia Clinical and Epidemiological Research Institute, Trujillo 13007, Peru; 4 Urology Department, Regional Institute of Neoplastic Diseases, Trujillo 13601, Peru

**Keywords:** fine-needle biopsy, cytology, capillarity

## Abstract

**Objective:**

To evaluate the diagnostic validity of fine-needle capillary cytology (FNCC) in palpable tumours.

**Material and methods:**

A retrospective, single-tray, cross-sectional diagnostic test study was carried out. We reviewed the cytological reports of the case files of the Cytology Unit of the Northern Regional Institute of Neoplastic Diseases (IREN) from January 2012 to December 2016.

**Results:**

A total of 332 patients were selected, with an average age of 54.77 years (range 13–90 years); 61.4% of patients were female. The most frequent anatomical sites were lymph nodes (49.7%), thyroid (13.3%), breast (12.3%) and soft tissues (11.4%). Twenty-five cytologies did not have a histological correlation and six showed an atypical result. In the lymph node study, the most frequent pathology was metastatic carcinoma (49.7%), followed by lymphoma (13.3%). The FNCC had a sensitivity of 99.55%, a specificity of 98.77%, a positive predictive value of 99.55% and a negative predictive value of 98.77%. The positive likelihood ratio was 80.63%.

**Conclusions:**

FNCC is a useful, safe, reliable and economical ambulatory technique with minimal complications and high diagnostic accuracy.

## Introduction

The origins of fine-needle aspiration (FNA) can be traced back to Arabic medicine [1, 2]. Centuries later, Kun (1847) described the technique and its benefits for diagnosing diseases, and this utility was confirmed by other authors in neoplastic and infectious pathology [2–5]. During the second decade of the 20th century, Dudgeon and Patrick in the United Kingdom and Martin and Ellis in the United States began the use of punctures for the rapid diagnosis of tumours [3, 6]. The first series of cases from the New York Memorial Hospital, in which an 18-gauge needle was used with good results [3, 7, 8], were published in the 1930s. At the same time in Europe, researchers such as Söderström and Franzen (Sweden), Lopes Cardozo (Holland) and Zajdela (France) termed the technique ‘*fine-needle aspiration cytology*’ (FNAC). Zajicek and Franzen at the Karolinska Hospital were the first pathologists to evaluate the diagnostic precision of the technique [3], popularising its use and coining the term ‘FNA biopsy’ when they successfully introduced 25–22 gauge needles and the aspiration gun [1, 7, 8]. In 1981, Zajdela modified the technique using the capillarity principle, creating the ‘*aspirationless fine-needle*’ variant (FNCC), in which sampling is performed using only a very thin needle and no syringe. Satisfactory results were achieved [2, 6, 9] and have been replicated in other series [7, 8].

FNCC is based on the capillary pressure exerted in a very slim tube and which is sufficient to keep cells attached to their lumen [3, 9]. Several studies have shown its usefulness in thyroid, lymph nodes, vascular tissues and some solid organs, except in breast and soft tissue tumours in which FNAC has proven slightly better [3]. FNCC offers multiple advantages, including being minimally invasive and ambulatory, fast results, low cost, high precision (when performed by trained and experienced staff), low risk of complications, less technology than surgical biopsy, sampling of multiple lesions in one session, and sampling of superficial and deep lesions with the support of imaging techniques [3]. Complications in the procedure are infrequent, with minimal haemorrhaging and spontaneous haematoma resolution [10]. There are exceptional reports of haemorrhaging, septicaemia, biliary peritonitis, pneumothorax and other complications [3, 10, 11] in deep lesions. Additionally, some aspects described in the past, but which are not currently considered, included the possibility of cancer cell dissemination, local tissue alteration with the consequent difficulty of histological diagnosis (capsular pseudoinvasion and pseudomalignant reparative reactions) [3], all highly unlikely effects given the fineness of the puncture needle.

The fine-needle biopsy appeared in Peru in the 1990s. Columbie and Somocurcio (Edgardo Rebagliati Martins Hospital, Lima) were the first to use the aspirative version in the study of thyroid tumours, publishing their results in 1996 at the National Congress of Pathological Anatomy [12]. Years later, the National Institute of Neoplastic Diseases began to use the technique irregularly in assisting surgeons, but more recently in 2007 Abad, Takahashi and Dyer formally began to use cyto-interventionism in oncology, the first cases being in the lymph node and thyroid gland. To date, other hospitals have implemented this technique but they essentially use FNAC [13–15]. FNCC came to Peru in 2011, with the contribution of Ricardo Bardales in the newly created cytology unit of IREN Norte, the reference oncology centre of northern Peru, whose scope of action includes approximately 9 million inhabitants [16], and which became the first hospital to use the technique successfully in different organs and initiated its dissemination nationwide [17].

The present study evaluates the diagnostic validity of FNCC in palpable tumours and is the first Peruvian article in this regard.

## Materials and methods

A retrospective, single-tray, cross-sectional diagnostic test study was carried out. The population consisted of patients with palpable tumours treated at the Northern IREN, Peru, in the period from January 2012 to December 2016. The inclusion criteria were patients with palpable tumours who were referred to the Northern IREN Cytology Unit; FNCC performed by the cytopathologist; compliance with the necessary criteria for performing the procedure (description and explanation of the technique, acceptance of the procedure and informed consent). Exclusion criteria were tumours on which the fine-needle biopsy was not performed in the Cytology Unit; lack of histological correlation in the palpable tumours that were punctured; severe coagulation disorders; and patients younger than 11 years old.

*The technique:* FNCC, which is physically based on the principle of capillarity [3], was performed using 27–23 gauge needles, a 10 cc. syringe for discharging the material, microscope slides and coverslips, and 96% ethanol as fixative. The procedure involves holding the needle directly with the fingers, and inserting it into the target tissue, moving it back and forth in various directions for several seconds (depending on the tissue cellularity and vascularity) and then, with the help of a syringe, discharging the material onto a microscope slide and spreading it with another slide. Fixation was immediate in all cases, the adequacy of the sample being assessed with toluidine blue. The average number of passes was three. PAP and H&E staining were used for the final cytodiagnosis. A cell block was not obtained.

*Diagnostic test:* The test to be assessed was FNCC cytological diagnosis, defined as the diagnosis resulting from studying the cells of a tumour lesion obtained using FNCC. The gold standard was histological diagnosis, defined as the product of the study on organ tissues punctured with capillary action or on the primary lesion (in the case of metastasis). ‘Insufficient’ was defined as the category in which cytological findings included haematic material and few inflammatory elements. ‘Atypical’ was defined as the category in which the cytomorphological findings did not permit a classification as benign or malignant (grey area).

*Data acquisition process:* The cytological reports from January 2012 to December 2016 from the Northern IREN Cytology Unit files were reviewed, and the medical record number, epidemiological data and cytodiagnosis were obtained from these. To obtain technique performance consistency, a pathologist (MAL) from the unit, who was trained and experienced in the procedure, collected and carried out the cytodiagnosis. Subsequently, the medical records were reviewed and the histological diagnosis was performed and recorded by the unit’s pathologists, who did not know the cytological result. On this basis, the eligible population matching the inclusion and exclusion criteria was selected. Data were collected by an expert researcher and co-author (JGO), who is not a pathologist. These data were stored in a Microsoft Excel spreadsheet, and processed using SPSS version 23 and Epidat 3.1.

## Results

The average age of the 332 patients was 54.7 years, with a range between 13 and 90 years. The female/male ratio was 1.59. 61.4% of the patients were female with an average age of 52 years. The average age of males was 59 years old. In line with international standards, the cytological result was grouped into four categories: positive (68.7%), negative (28%), atypical (1.8%) and insufficient (1.5%). The histological diagnosis to assess the diagnostic accuracy of the technique was grouped into four categories: positive (66.9%), negative (25.3%), atypical (0.3%) and without histology (7.5%). The most frequent anatomical sites were lymph node (49.7%), thyroid (13.3%), breast (12.3%) and soft tissue (11.4%) (Table 1).

**Figure 1.**
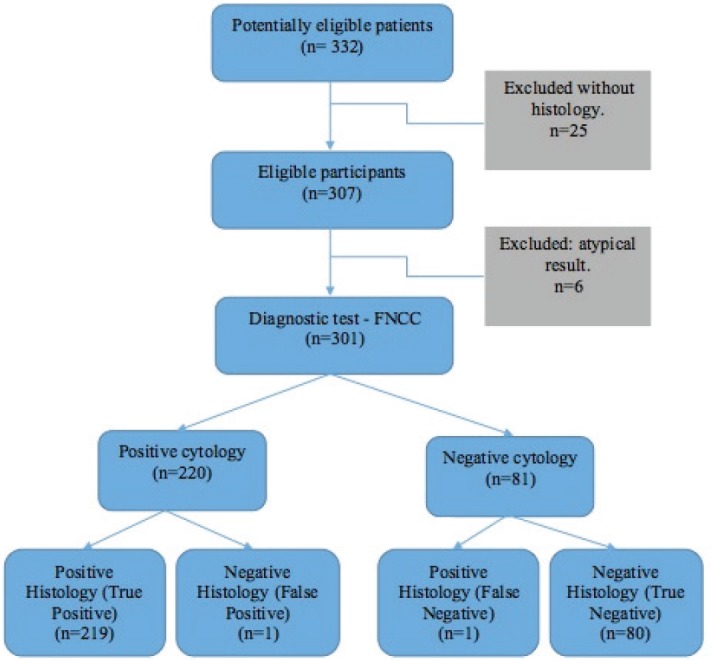
FNCC flow chart.

Of the 332 FNCC cases, 25 did not have histological correlation and six showed an atypical result. Consequently, the diagnostic test was carried out on 301 cases (Figure 1): 1.8% (5 cases) of the cytological findings were insufficient, corresponding to 1 thyroid sample, 2 inguinal node samples, 1 supraclavicular node sample and 1 axillary node sample (Table 2).

**Figure 2.**
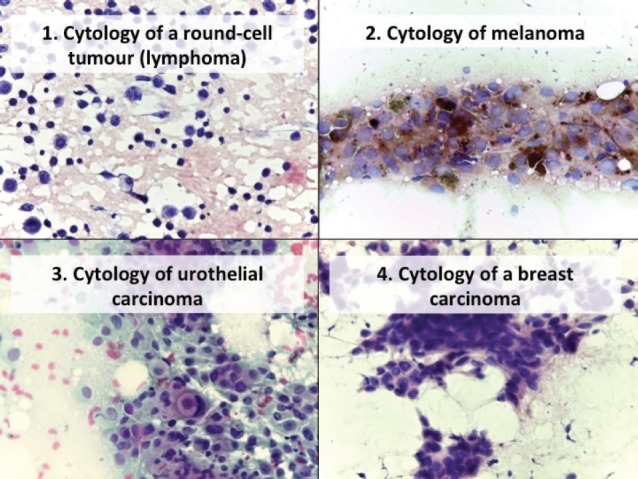
Cytology microphotographs obtained by FNCC.

**Figure 3.**
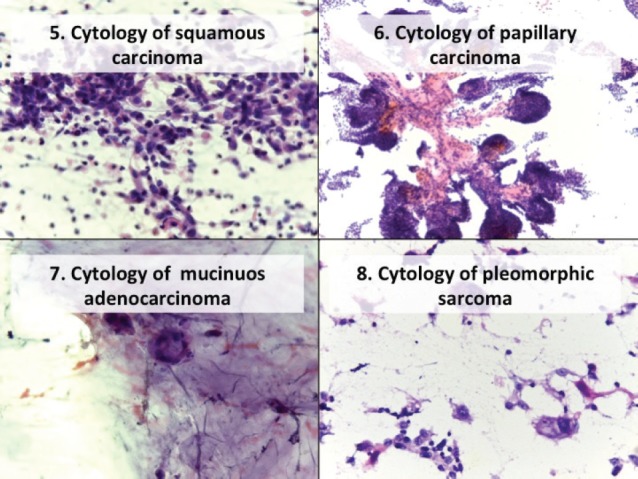
Cytology microphotographs obtained by FNCC.

In the atypical results, there were three lymph nodes (retroauricular and cervical), one orbit, one thyroid and one soft tissue (cervical region). Of these, three showed positive malignant histology (non-Hodgkin’s lymphoma, histiocyte-rich Hodgkin’s and insular carcinoma), two were negative (inflammatory pseudotumour and chronic, non-specific lymphadenitis) and one retained the histomorphological characteristics of atypical disease (Table 3). Comparing the type of tumour, the cytological diagnosis of adenocarcinoma presented a discordant histological result (carcinoma), while the cytological diagnosis of carcinoma showed three different histological findings (adenocarcinoma, lymphoma and melanoma). The cytological diagnosis of poorly differentiated sarcoma had a different histological correlation from poorly differentiated carcinoma (Figures 2 and 3).

In the enlarged lymph nodes studied, the most frequent diagnosis was metastatic carcinoma (49.7%), followed by lymphoma (13.3%) and metastatic adenocarcinoma (12.7%). Of the 114 cases of metastatic lymph node disease, the most common primary cases were breast, stomach, prostate, thyroid and skin.

For the diagnostic test analysis, 301 cytological samples fulfilling the eligibility criteria were selected (Table 4), excluding the insufficient cytological results that did not have histological correlation. The false positive result was a round-cell malignant tumour, whose histology reported lymphoid hyperplasia, whereas the false negative result was an inflammatory cytology whose histology reported a lymphoma. FNCC had a sensitivity of 99.55%, a specificity of 98.77%, a positive predictive value of 99.55% and a negative predictive value of 98.77%. The positive likelihood ratio was 80.63%.

## Discussion

Fine-needle cytology is a simple technique with high diagnostic value and growing interest worldwide [11, 18]. The FNCC variant is a safe, simple and quick, minimally invasive technique that can be performed on outpatients. It is economical and reproducible and has a high diagnostic accuracy. Its use has been demonstrated in the treatment of various diseases, particularly cancers [19–21]. The use of the technique in Peru is recent and not widespread and therefore no reports have been published about it. In this study, 332 patients with palpable tumours permitting the use of FNCC were assessed. The average age was 54.55 years, slightly under the age at which patients present with oncological diseases.

The largest number of lesions was in lymph nodes, demonstrating the usefulness of FNCC in lymphadenopathy and its advantages over FNAC, as reported by Sajeev *et al* [22] and Farooq *et al* [23]. The high number of lymph node tumours is probably due to the fact that IREN North is a national reference centre in oncology, and therefore patients come not only for diagnosis but also for follow-up. Metastatic lymph node disease represented the highest percentage, similar to what was reported by Zhou *et al* [24] and Anila *et al* [25], breast cancer being the most common. In our region, cancer among women is a serious public health problem, because the vast majority of women only come to the hospital once they are in the advanced stages. Second, we find the primary nodal disease, reported cytologically as a round-cell malignant tumour, which, in the histological and immunohistochemical study, corresponded mainly to diffuse large B-cell lymphoma. In our findings, we should mention the group of benign diseases, in which granulomatous lymphadenitis stands out, similar to what was reported by Bharathi *et al* [7, 26]. These results are of epidemiological interest, for consideration in the differential diagnostic approach (pseudotumour pathology).

Thyroid disease was the second most frequent disease assessed using FNCC, with good results. On this point, the American Thyroid Association states that FNA biopsy is a very important method in the assessment of thyroid nodules and recommends its use in nodules larger than 1.0, 1.5 and 2.0cm, depending on whether the ultrasound malignancy risk is intermediate, low or very low, respectively [27]. This document mentions FNA, however, in literature there are several works that validate the utility of FNCC, with results similar to our study [20, 28–31]. The work of Zhou *et al* suggests that the size of the lesion could be used as a reference for choosing which technique to use [32]. In recent years, in Peru, the use of FNCC in the thyroid has increased, due to the reliability of the procedure.

Other assessed diseases were tumours of the head and neck including tumours of the eye and conjoining tissue, with satisfactory results, similar to those reported by Hamaker *et al* [33], Braun *et al* [34] and Deshpande *et al* [35]. Hamaker’s study on masses in the head and neck reports that, although FNCC produces lower-volume samples, the quality is good and the cell architecture is preserved, ensuring a high value diagnosis [33]. Two years later, Braun’s group compared 166 lesions in the same anatomical region and found that FNCC was more effective in the thyroid, lymph nodes, thyroglossal cysts and salivary glands, with the exception of the submaxillary salivary glands [34].

Regarding diagnostic accuracy, using the described, internationally accepted categories, the technique achieves values that reflect high reliability and does not differ significantly whether or not ultrasound is used [36–38]. The use of capillary cytology in the context of suitable clinical and imaging context is an accurate diagnostic tool [33, 36]. Our results are similar to those described by other authors, ranging from 92% to 99% depending on the organ studied [19, 22, 25, 39]. Lower percentages of diagnostic accuracy have been described in the series by Tauro *et al* [20] and McElvanna *et al* [40].

In a separate comment, it is worthwhile comparing FNCC and FNAB, which both use a ‘fine needle’, a very thin needle that, regardless of the physical principle with which it is used, allows cells to be extracted from a given mass [41]. The diagnostic accuracy of both is operator-dependent and therefore requires proper training, sensitivity and high specificity for malignancy, not only for diagnostic purposes but also for therapeutic and prognostic purposes [11, 30, 42]. The main technical difference between FNCC and FNAC is that the former eliminates the handling of the device, using only the needle, thus making the technique simpler with equal or better diagnostic accuracy in certain pathologies [21, 31, 43, 44]. It has also been shown that with a smaller needle diameter, a better quality of cell material is obtained, while at the same time reducing patient discomfort and the risk of bleeding [45]. Another important aspect is the immediate fixation of the slide in 96% ethyl alcohol to prevent desiccation from affecting the cellular details and hindering the diagnosis [26, 40, 43, 45]. Furthermore, this procedure is readily accepted by the patient, who does not require anaesthesia, and can be repeated if the sample was inadequate (immediate evaluation) [26, 46, 47]. Madhuri *et al* (1998) used FNCC to assess 670 patients with superficial and deep lesions in various organs, using imaging for the deep lesions, and reported high diagnostic accuracy, highlighting the fact that this method allows greater technical control and a better perception of tumour consistency [19]. In the opinion of Tauro and De Carvalho, given that differences are not statistically significant between the two techniques, the choice of one over the other will depend on the operator [20, 39], who must be adequately trained [14, 42].

Among the limitations of the technique is the differentiation of some types of malignant lesions, such as poorly-differentiated tumours, which, even in a histological correlation, are not adequately typified without the help of an immunohistochemical study, as well as the definition of invasive or *in situ* tumours, which are determined by studying the tissue itself [26, 48]. On this point, we note that immunocytochemical tests have been carried out in recent years, not only to determine cell lineage but also for prognostic purposes, despite the poor amount of material that is obtained. Some alternatives such as thin-layer cytology and the use of the cell block have been suggested with promising results [48–50]. In the present study, we did not include this method when it was carried out in the surgical biopsy. Finally, FNCC is an effective alternative which, due to its benefits, should be considered in public health policies within the context of a diagnostic approach to a cancer patient, in view of the cost and waiting time that a histological diagnosis of a surgical biopsy entails in many hospitals.

## Conclusions

FNCC is an ambulatory, fast-diagnosis, economical and reliable technique with minimal complications that must be carried out by trained staff.

The diagnostic accuracy of FNCC is high, and because of its advantages, should be promoted and implemented in the country and in other regions that have a similar situation to Peru.

## Conflicts of interest

The authors have no conflict of interests to declare.

## Authors’ contributions

The main idea came from MAL. JGO and MAL collected and analysed the data. MAL, JGO, JCA and JDP wrote the protocol and manuscript and approved the final version.

## Figures and Tables

**Table 1.** FNCC: epidemiological characteristics.

	*N*	*%*
**Total cases**	332	100
**Average age (years)**	54.77	–
**Female/male ratio**	1.59	–
**Gender**		
- Female	204	61.4
- Male	128	38.6
**Anatomical site**		
- Lymph node	165	49.7
- Thyroid	44	13.3
- Breast	41	12.3
- Soft tissues	38	11.4
- Salivary gland	16	4.8
- Eye and connected tissues	12	3.6
- Skin	6	1.8
- Paranasal sinuses	5	1.5
- Oral cavity and tonsils	5	1.5
**Cytological diagnosis (test)**		
- Positive	228	68.7
- Negative	93	28.0
- Atypical	6	1.8
- Insufficient	5	1.5
**Histological diagnosis (gold standard)**		
- Positive	222	66.9
- Negative	84	25.3
- Atypical	1	0.3
- Without histology	25	7.5

**Table 2.** Fine-needle capillary cytology: cytohistological correlation.

		Histological diagnosis			
		Positive	Negative	Atypical	Without histology
Cytological diagnosis	Positive	219	1	0	8
	Negative	1	80	0	12
	Atypical	3	2	1	0
	Insufficient	0	0	0	5

Source: Data obtained by the researchers

**Table 3.** FNCC: diagnostic correlation of the cytological and histological results.

		Histological diagnosis							
		Adenitis	Atypical	Adenocarcinoma	Carcinoma	Lymphoid hyperplasia	Lymphoma	Melanoma	Inflammatory pseudotumour
Cytological diagnosis	Atypical	1	1	–	1	–	2	–	1
	Adenocarcinoma	–	–	29	1	–	–	–	–
	Carcinoma	–	–	1	142	–	1	1	–
	Inflammatory cytology	–	–	–	–	–	1(FN)	–	–
	Malignant round-cell tumour	–	–	–	–	1(FP)	27	–	–
	Sarcoma	–	–	–	1	–	–	–	–

Source: Data obtained by researchers

FN: False negative

FP: False positive

**Table 4.** FNCC: diagnostic test results.

	Gold Standard: Histology		
	Malignant	Benign	Total
Diagnostic test: FNCC	Positive	219	1
	Negative	1	80
	Total	220	81
	Value	IC (95%)	
Sensitivity (%)	99.55	98.43	100
Specificity (%)	98.77	95.74	100
Validity index (%)	99.34	98.25	100
Positive predictive value (%)	99.55	98.43	100
Negative predictive value (%)	98.77	95.74	100
Prevalence (%)	73.09	−	−
Likelihood ratio (+)	80.63	−	−
Likelihood ratio (−)	0	−	−

Source: Data obtained by the researchers
